# Should GPs ask patients about their financial concerns? Exploration through collaborative research

**DOI:** 10.1080/02813432.2020.1753344

**Published:** 2020-04-16

**Authors:** Solveig Osborg Ose, Live Standal Bøyum, Silje Lill Kaspersen, Arman Vestad, Per-Arne Gjelsvik

**Affiliations:** a SINTEF, Health services research, Trondheim, Norway;; b OsloMet, SIFO, Oslo, Norway;; c Labour and welfare services (NAV), Trondheim, Norway;; d NAV, Røros, Norway

**Keywords:** General practitioners, financial concerns, mental health, financial counselling, collaborative research

## Abstract

**Objective:** Health services should arguably be concerned about the financial situation of patients since health problems can cause financial concerns, which in turn can cause health problems. In this study, we explored the role of the general practitioner (GP) as a potential early discoverer of financial problems who can refer at-risk patients to financial counselling services.

**Design:** A collaborative health service research experiment. For four weeks, GPs asked their patients predefined questions about financial concerns and health, by anonymous data mapping. GPs shared their experiences with the researchers after the experiment.

**Setting:** One GP office in Norway.

**Subjects:** A total of 565 patients were included in data mapping by 8 GPs.

**Main outcome measures:** Patient prevalence data and GPs experimental data of patients’ health problems that caused financial concerns and financial concerns that affected patients’ health.

**Results:** Of 565 GP patients, 11% (*n* = 63) indicated that they had health problems causing them financial concerns, or vice versa; 9% of patients reported health problems causing financial concerns and 8% of patients reported financial concerns that affected their health. Through the data mapping experiment GPs became aware of financial concerns of their patients and by this expanded and improved their therapeutic toolbox. Several months after the experiment the GPs reported that more patients received financial counselling since the GPs asked their patients about financial problems more often than before and because the patients had heard that GPs cared about such problems.

**Conclusion:** Our results suggest that GPs can be early discoverers of financial problems interacting with their patients’ health. When there are no clear medical explanations for the health problems that prompted the consultation, the best therapy may thus be financial counselling.

**Key points**
- Many people live on the edge of financial ruin and struggle to keep track of their finances, but limited research exists that investigates associations between finance and health.
- In a collaborative health services research experiment 11% of the patients at a Norwegian GP office had health problems that caused them financial concerns, or vice versa.
- GPs found it helpful to ask patients about their financial concerns when no clear medical explanations for their health problems was found. Then free financial counselling services could be offered.

## Introduction

Many people live on the edge of financial ruin and struggle to keep track of their finances; however, little evidence-based research on associations between finance and health exists [1].

The individual financial consequences of health problems depend on where individuals live. Important factors include insurance coverage, health service availability, access to support, scope and effectiveness of prevention programmes, level of disability, and family and community networks [2].

On the other hand, financial problems can also cause various health problems, especially mental health problems [3,4]. Both low incomes and debt are associated with mental illness, but it has been suggested that the effect of income may be strongly mediated by debt [5]. A systematic review of the long-term consequences of indebtedness on health has demonstrated serious health effects related to indebtedness [6]. This study found that individuals with unmet loan payments had suicidal ideation and suffered from depression more often than those without such financial problems, and that unpaid financial obligations were related to poorer subjective health and health-related behaviour. The authors suggested that debt counselling and other programmes to mitigate debt-related stress were needed to alleviate the adverse effects of indebtedness on health. Others have suggested implementing socio-economic programmes that address and prevent hardship to promote community mental health [7]. Another study concluded that efforts to improve consumer financial literacy should be supplemented by programmes designed to increase consumer involvement in financial counselling because this combination is found to foster co-production and improve consumers’ financial well-being [8].

According to figures from the Financial Supervisory Authority of Norway, consumer debt in Norway is now over NOK 100 billion and the growth of such debt has accelerated significantly in recent years. The rate of default on consumer loans is generally higher than that on other loans from banks and finance companies, and there has been an increase in defaults. High profitability over a long period of time has made consumer loans an attractive product for new providers [9]. According to the Ministry of Children and Equality in Norway, young people especially have problems with consumer debt, and the ministry has emphasized that debt problems can destroy private finances, resulting in broken families and health problems [10].

Norway is part of the Nordic welfare model with universal health care and a comprehensive social security system. The local authorities – i.e. the municipalities – are responsible for providing financial advice to people with payment or debt problems. This is a statutory service that in most municipalities is managed by the local labour and welfare office. However, financial counselling is typically utilized at a very late stage in the individual debt accumulation process; therefore, local labour and welfare offices must often handle very complex cases involving many creditors. The counsellors find some service users coming to counselling carrying plastic bags full of unopened bills when many resources are required to solve their problems. The complexity of the cases means that only a small number can be managed. Accordingly, the Norwegian Consumer Council has revealed that the population in many municipalities must wait a long time to access this statutory service [11]. Financial counsellors that provide the service argue that there are major prevention opportunities if they could provide advice and counselling at an earlier stage before the cases become too complex. They have suggested that general practitioners (GPs) could identify financial problems at an earlier stage and establish contact between the person in need and the financial counsellor at the local labour and welfare office. Employers could also connect people with financial problems with the financial counselling service.

This explorative study is about the GP as a potential early discoverer of financial problems who can refer at-risk patients to the local financial counselling service. The research questions are (a) Is the GP a suitable early discoverer of individuals with financial problems? (b) Should the GP ask their patients about their financial concerns?

## Method

To obtain more knowledge about the potential role of GPs in preventing the negative health effects of people’s financial concerns, a collaborative health service research approach was undertaken. Collaborative research projects have emerged as a particular form of academia–industry interaction involving systematic documentation and publication of results [12]. In such projects, the practitioners can play the role of informants, recipients, endorsers, commissioners or co-researchers [13]. Our research plan included the following steps: 1) establish trust and robust personal relationships between main stakeholders, 2) obtain funding for a collaborative health service research experiment, 3) involve GPs in the experiment to explore the potential for GPs to be early discoverers, and 4) obtain and analyse data, then report the results.

The collaboration was initiated by the person responsible for the professional development of debt counselling in the labour and welfare services in a national project (author AV) and began with several informal meetings with the researchers (authors SOO, LSB, SLK). The collaboration with a municipality with one GPs’ office (eight GPs with about 6000 patients on their lists) was previously established by the labour and welfare services, and a more formal meeting with one senior GP from the office who also had a position as a chief physician at the local labour and welfare office (author PAG), the manager of the GPs’ office, the manager of the local labour and welfare office and a financial counsellor from the local labour and welfare office. In another meeting, the researchers met with the GPs one evening to plan the experiment.

The team obtained funding to conduct a small experiment from the Norwegian Research Council (grant number: 281198). In this experiment, for four weeks during the spring (April 3–May 4) of 2018, the eight GPs asked their patients several predefined questions about their financial concerns and health. Anonymous patient data were collected to obtain prevalence data and the GPs shared their experiences with the researchers after the experimental period.

### Setting

Every resident of a Norwegian municipality is entitled to be registered as a patient with a primary physician according to the Regular GP Scheme. The Regular GP Scheme is voluntary, but only 0.4% of the Norwegian population have chosen to remain outside it. A regular GP is responsible for examining, diagnosing and treating the patients on his/her list, and this includes prescribing medication and issuing medical certificates. The regular GP is also responsible for referring patients to hospitals and other medical specialists. The patients pay a relatively low consultation fee when visiting the GP unless they have an exemption card.

### Design: collaborative health service research experiment

The goal of the experiment was to collect data on (a) the prevalence of patients experiencing health problems that lead to economic difficulties, (b) the prevalence of patients with financial concerns leading to health problems, and (c) GPs’ experiences of asking their patients about their financial concerns and what they learned from the experiment.

We used a complex mixed-methods design [14] using qualitative methods to establish the collaboration, then a quantitative method to obtain prevalence figures without collecting sensitive data (the experiment). After that, we used further qualitative methods in relation to a focus group discussion with the GPs after the experiment where they shared their experiences of conducting the experiment. Notes from the focus group discussion were analysed thematically [15].

#### Planning and setting up the experiment

No information about the identity of the patients was needed; therefore, patient data collection could be conducted completely anonymously. The eight GPs were to ask their patients six questions and collect their answers in an anonymous external database that was not linked to the patient record system. The team agreed on the following exclusion criteria: patients younger than 18 years of age, patients who did not understand Norwegian, patients with no welfare rights in Norway, patients in acute need of medical help, and patients with permanent reduced cognitive functioning (cognitive impairment) were excluded from the study. The six questions that were included in the mapping are shown in Table 1.

**Table 1.** Questions and response categories included in the mapping of patients by the GPs.

Topic/question	Response categories
(i) Sex	Male/female
(ii) Age	Seven categories
(iii) Do you have health problems that cause you financial concerns?	Yes/no, don’t know, prefer not to answer
(iv) Do you have financial concerns that cause health problems?	Yes/no, don’t know, prefer not to answer
(v) Do you want to speak with a financial adviser in the labour and welfare services?	Yes/no
(vi) If yes, what type of financial counselling do you want?	Financial counselling from the local labour and welfare office, I would like to talk anonymously to a national financial counsellor phone service (see appendix), I don’t know

Eight tablets were purchased, and the electronic survey was set up using a technical safe solution provided by the University of Oslo.

Prior to the experiment, the project team produced several media initiatives for both local and regional media (newspapers and radio) to prepare the population for the experiment. A formal announcement was published in the local newspaper in the print edition. An information poster was put in the waiting room of the GPs’ office. The GPs received written information sheets from the local labour and welfare office with contact information for financial counselling to be handed out to patients.

#### Unexpected events during the experiment

On the second day of the experiment, there was critical input in the form of a reader’s letter in one of the local newspapers, expressing feelings of scepticism and confusion regarding the experiment at the GPs’ office. The project team replied to the letter the same day, including the questions that GPs were to ask their patients. The mapping recommenced after two days, and it was now unproblematic for the GPs to ask their patients the six questions. Therefore, the critical letter helped to resolve uncertainties and the rest of the mapping was conducted without problems. The GPs said afterwards that the inclusion of the questions in the reply rendered the experiment harmless and reassured potential respondents, who were not sceptical about the experiment when they arrived at their consultations. In retrospect, we realized that the questions should have been communicated more clearly *via* the media before the experiment started. Another lesson was that such communications should not be placed behind paywalls in the newspapers. Nevertheless, the GPs found that almost every patient had read about the project and was prepared for questions about their financial concerns when they came to their consultations.

### Ethics

Social science experiments collecting anonymous data in collaboration with health and labour and welfare personnel do not require pre-approval from the data protection authorities in Norway. However, the researchers responsible for the experiment consulted the data protection authorities when planning the experiment. No audio recordings were made of the meetings between GPs and researchers prior to mapping or of the interview conducted after the survey.

## Results

### Results of patient mapping

A total of 565 patients were included in the mapping. According to estimates of the total number of consultations at the GPs’ office during the experiment period, this amounted to 75% of all consultations with patients over 18 years of age. Based on population data from Statistics Norway, we found that women were slightly better represented (15%) than men (11%), except for the two oldest age groups where men were slightly better represented than women, i.e. 20% for men and 16% for women. In general, representativeness increases with age.

**Figure 1.**
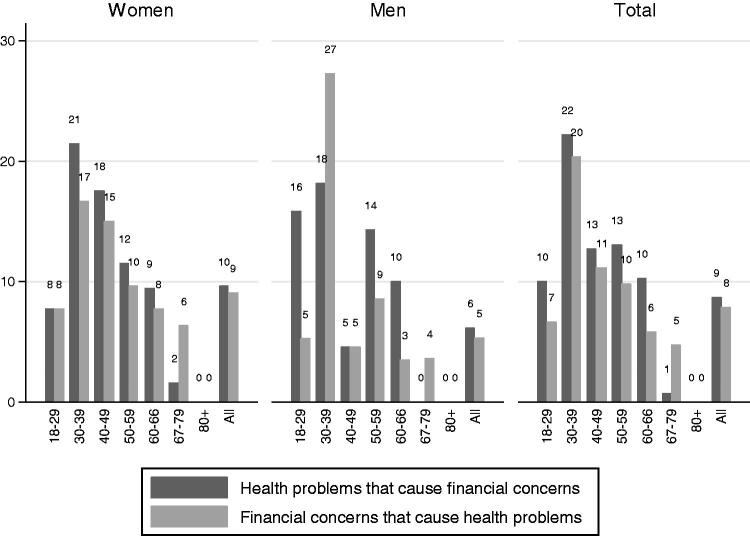
Percentage of the patients who answered yes to the following questions: (a) Do you have health problems that give you financial concerns? (b) Do you have financial concerns that affect your health?

The answers to the two main questions are shown in Figure 1. In total, 10% of women and 6% of men reported health problems that caused them financial concerns. A smaller proportion, 9% of women and 5% of men, reported financial concerns that affected their health.

The highest prevalence of health problems causing financial concerns was found among patients in the 30–39 age group, and as Figure 1 shows, men in this age group were especially under-represented. However, those who had one or more consultations with their GPs (or those included in the mapping) had a high prevalence of financial concerns causing health problems, and 27% of the men in the 30–39 age group reported such problems.

About 5% (*n* = 30) of the patients answered in the affirmative to both questions, and 11% (*n* = 63) of the patients answered that they either had health problems that caused them financial concerns or financial concerns that affected their health.

In addition, 3% (*n* = 16) of the patients answered that they had no health problems that gave them immediate financial concerns, but that they were at risk in the future; 2% (*n* = 9) answered that they did not know and 1% (*n* = 6) did not want to answer the question of whether they had health problems that caused them financial concerns. Overall, 86% (*n* = 485) answered that they did not have health problems that resulted in financial concerns.

For the second question, 3% (*n* = 19) answered that they did not know whether they had financial concerns that affected their health situation and 1% (*n* = 4) did not want to answer. Overall, 88% (*n* = 495) answered that they had no financial concerns that affected their health.

Those who answered yes to one or both of the questions were asked whether they wanted to talk to an economic adviser at the labour and welfare office.

Twenty-eight per cent of those with financial concerns responded that they wanted to talk to an economic adviser in the labour and welfare services. Furthermore, these were asked about the type of financial advisory service they wanted. More than half (55%) wanted to speak with a financial counsellor at the local labour and welfare office, while 17% wanted contact with an available anonymous national telephone advice service. The remaining 28% responded that they did not know who they wanted to contact.

### The GPs’ experiences

#### Experiences of the experiment

In general, the GPs found it easy to ask patients about their financial concerns and reported that the exclusion criteria worked well. They believed that the patients answered the questions honestly and that no patient relationship became worse because they asked these questions. Some of the patients were also relieved to be able to talk about their financial concerns. There is much humour in the local population, and one of the patients appeared with a bank statement to prove that he had good control of his private finances, while another slapped an old paper bankbook on the GP’s desk. Several of the GPs thought that asking about financial concerns in the experiment helped them realize the importance of addressing this topic in appropriate cases. They thought that this was an easier subject to address than topics such as alcohol consumption. They also explained that they frequently asked patients much more intimate questions than those about financial concerns, but they emphasized that this question, like all the others they asked their patients, had to be used with knowledge and understanding in the right situations.

#### No clear pattern

Before the GPs saw the results of the mapping, the researchers asked whether they believed it would be women or men who had the most financial concerns. Some of the GPs thought it would be mostly women, while others had no clear expectation about the sex distribution. They generally believed that quite a few of the patients would report financial concerns.

#### Most cases were known

The researchers asked the GPs whether they typically knew in advance which patients would answer ‘yes’ or ‘no’ to the questions. The most experienced GPs responded that they typically had this information about the patients before they asked. Nevertheless, those who were relatively new to the job found that they could generally tell who would answer ‘yes’ before they asked. This meant that they often evaluated the patient’s life situation in general, especially when the patients had mental health and/or substance abuse problems. In a few cases, the GPs received new knowledge about patients’ life situations and were uncertain whether the patient would have shared this information unless explicitly asked about financial concerns. Many of the patients who responded affirmatively to one or both main questions were already in contact with the local labour and welfare office, but not necessarily with the financial counsellors. However, in some cases, the patient was not in contact with those services but wanted to contact them when they learned of the financial counselling there. In line with the results from the patient mapping, some also wanted to use the anonymous advisory service.

#### Some have low confidence in labour and welfare services

The GPs also regularly met patients with low confidence and trust in the labour and welfare services. Some GPs found that some patients were afraid that the GPs operated as ‘agents’ and feared that the GPs wanted to report the patient’s responses to the labour and welfare office. There were also patients who reported having answered the questions for the GP’s sake and not for the sake of the labour and welfare office. The GPs emphasized after the experiment that it was important for them not to act on behalf of the labour and welfare office because people’s trust in their GPs could be weakened.

#### GPs did not reach the younger patients

The discussion also covered young people with consumer debt. The GPs responded that those in this group were typically not in contact with their GPs because they have less frequent health problems. For example, many young people in the municipality left secondary school without completing it, and these individuals could be at risk of accumulating unmanageable consumer debt. If they did not yet have a health problem or health consequences from the financial strain, the GP would have no contact with them. The impression of the GPs was that this largely concerned young men rather than young women.

However, several young people with permanent functional disabilities lived in the municipality, and the GPs believed that they often had better finances than disabled adults or other young people. We also discussed whether people with disabilities were prone to building high levels of consumer debt. However, we were unable to draw a conclusion because there were insufficiently rich financial data available to study this issue.

#### When do people experience financial problems?

The GPs agreed that the vast majority of those who suffered serious health problems and received disability insurance largely adjusted their consumption when their income was permanently reduced. In Norway, a person on a disability benefit receives about two-thirds of their previous income. The GPs explained that to live with a chronic disease, the patients needed good structure in their lives because there was much to follow up both inside and outside the health services. Those with chronic and prolonged somatic disorders often had a good structure in their lives and in relation to their financial situation. However, the GPs were more concerned with patients with other issues, such as gambling addictions, substance addictions and mental illnesses. They suggested that these patient groups were more at risk of losing control of their private finances, perhaps precisely because it was difficult to keep structure in their lives owing to their mental problems. The GPs also observed that psychiatric services had severe capacity problems and that many patients waited for a long time to receive help and often received no help at all. These patients were assumed by the GPs to have a high risk of accumulating significant debt and a high risk of ending up with plastic bags full of unpaid bills at the labour and welfare office – i.e. outstanding balances accumulating into unwieldy levels of debt.

Reflecting on other patient groups at risk of over-indebtedness, the GPs mentioned families who moved around different municipalities because they were afraid of the local child welfare services. The GPs’ experiences were that in cases involving substance abuse, the child welfare service had good methods and routines, while it was more difficult to come to terms with families when mental disorders were an important part of the picture. These families moved when the local child welfare services started to show interest. When they moved to a new municipality, they had a period of freedom until public child services (including kindergartens, schools, child and youth services and child welfare services) discovered the situation, and they moved again when someone started asking questions. The GPs suggested that these families may be at risk of accumulating expensive consumer debt.

#### Are GPs suitable actors?

One of the GPs had a patient who clearly stated that the GPs were not the appropriate actors to ask for private financial information because the GPs had high wages. However, the GPs agreed that they could address the issue of financial concerns if they had somewhere to send the patient for help. They had to be confident that the labour and welfare office would follow up and could provide good financial counselling and guidance for the patient. They also emphasized that it was not natural for the GPs to encounter people before they had health problems, when it may be too late to avoid severe financial problems. The GPs learned from the experiment that they did not detect many who were early in the process of accumulating consumer debt.

All GPs considered that asking about financial concerns should not be a formal task of GPs because it was not always a natural part of the consultation. Obviously, it seemed inappropriate to ask about this when only a cell test was conducted or when someone needed to be treated for acute injuries. However, when medical certificates are required or a situation arises that causes drastic changes in the life situation, it may be natural to address concerns about personal finances.

The GPs explained that after the experiment they were generally more able to identify patients who should be asked about financial problems. If the patient was tired, perhaps having sleep problems or depression symptoms, they might ask. However, if the patient had a bone fracture or another specific health issue, the topic would certainly not be relevant.

The GPs also stressed that it was important to remain aware that general practice differs from specialist care in that it focuses on the health of the whole person, combining the physical, psychological and social aspects of care. Financial concerns are within the social aspect, and one of the GPs argued that they should refer patients to social services in the same way they referred patients to hospitals and other medical services.

The researchers also asked the GPs whether they believed that employers could help the financial counsellors at the labour and welfare office to identify severe cases earlier. The GPs’ responses were unanimous: they considered that it would be more intrusive for the employer than the GP to address this topic.

Several months after the experiment, the GPs involved reported that more patients were now receiving financial counselling from the labour and welfare office than before the experiment. This is because the GPs asked their patients about financial problems more often than before and because the patients had heard or read that the GPs at this office cared about such problems.

## Discussion

In this study, eight GPs asked their patients predefined questions about financial concerns and health for four weeks and anonymous patient data were collected. Of 565 patients, 11% (*n* = 63) indicated that they had health problems causing them financial concerns, or vice versa; 9% of patients reported health problems causing financial concerns and 8% of patients reported financial concerns that affected their health. Through the data mapping experiment GPs became aware of financial concerns of their patients and by this expanded and improved their therapeutic toolbox.

As an attempt to bridge the gap between research and practice, interdisciplinary, collaborative and partnership research is requested [13]. Primary care must manage the health care of the patient holistically with all his or her complex needs, and research collaboration between specialists and primary care can provide new knowledge [16]. The GPs are key collaborators in the health-care system [17]. In this study, we established a collaborative research team that includes the local GPs’ office with eight GPs, the local labour and welfare office and applied social science researchers. The initiative was undertaken by the person responsible for the professional development of financial counsellors in labour and welfare services at the national level. The experiment was for a period of four weeks during which the eight GPs asked their patients several predefined questions about their financial concerns and health. The GPs recorded the answers from the patient in an anonymous external database that was not linked to the patient record system. After the experiment, a workshop was held where the GPs shared their experiences of the experiment with researchers.

The patient mapping in the experiment showed that 11% of the patients either had health problems that caused them financial concerns or/and had financial concerns that affected their health. We do not have comparable prevalence figures from population studies, but it has been found that 7% of households in Norway had recurring payment problems [18]. This accounts for 160,000 households and 355,000 people, and we calculated that this amounts to about 9% of all people between 18 and 80 years old. However, because the current study is about financial concerns and Poppe [18] asked more specifically about recurring payment problems, these prevalence figures are not exactly comparable.

The GPs seemed to reach part of the at-risk population, but with lower coverage of men than of women, and lower coverage for young people than older people. They probably do not reach those most at risk of financial problems. Lower representation of men in the GP population is in line with previous research [19–21] and with research on younger people’s health-seeking behaviour [22,23].

The lower representation of young age groups in the GP population may partly explain why the GPs did not discover many patients who were early in the debt accumulation process. Moreover, this may be related to the fact that health problems and contact with the health services do not appear until later in the debt accumulation process. When health problems as a result of the economic problems become apparent, the financial problems have probably persisted for some time.

Others have previously argued that psychiatrists should know how to respond when a patient reports a ‘debt crisis’ and look for signs that their patient could be at risk of debt (crisis prevention), raising the subject and discussing it with patients (including during routine assessments), effectively referring individuals for specialist debt counselling (and monitoring progress). They should assess whether patients have sufficient mental capacity to manage their finances or assign control of their finances to external sources (appointees and attorneys) [24]. Many of the same arguments could apply to GPs, and maybe especially that they should refer the patient to specialist counselling because this may be as important for the patient’s health as referral to specialist medical or mental health services.

The experiences from the experiment imply that it is not very difficult for the GPs to ask their patients questions about financial concerns because they often deal with much more intimate issues than financial concerns. From the international literature, we find that GPs in the UK engage with patients about a variety of social issues in addition to direct clinical work (‘non-health’ issues), such as health‐related benefits and debt, so there have been initiatives to collocate general practice with welfare advice services [25]. In Norway, the local labour and welfare office provides financial counselling, and it needs assistance to position itself to give financial counselling earlier in the debt accumulation process. It is at least important for GPs to know that such a service exists, and they can refer their patients in need to it.

The rationale behind asking about the patient’s financial situation is that this could prevent the patient ending up with very poor mental health owing to debt accumulation by referring the patient to financial counselling at an earlier point before the debt burden becomes unwieldy.

This is similar to the alcohol debate, where primary care has been promoted for decades as the key setting for delivering brief individual advice and counselling interventions to reduce heavy alcohol consumption. It is now considered unlikely that brief interventions alone confer any population-level benefit, and that their ultimate public health impact will derive from working in concert with other effective alcohol policy measures [26]. The same might be the case for other social and personal problems, including financial problems.

A recent systematic literature review showed that job losses, debt and financial difficulties were associated with an increased risk of mental illness and suicide in the general population and that interventions targeting people in debt or unemployment may reduce these effects [27]. The Norwegian survey of the general population about the causes of reoccurring payment problems showed that the most frequently mentioned reason is that one loses an overview of private finances (39%) [13]. Other frequently mentioned reasons in this survey were unemployment (35%), illness (27%), break-ups (19%), moving to new accommodation (8%), becoming a pensioner (8%) and other unspecified reasons (30%). As noted by Poppe [18] and others [28,29], the relationship between payment problems, income and debt is complex.

The GPs in this experiment did not generally find that financial problems arose when there were major changes in the patient’s life, e.g. becoming disabled or unemployed. The GPs found that most people whose income was reduced by unforeseen life events were likely to succeed in reducing their consumption and expenditure. However, those who did not have a health issue at the same time did not consult the GP. The labour and welfare services are probably in a better position to identify at-risk individuals because they know when people are unemployed and when people retire from work. These groups should be targeted for financial counselling, as these are situations in which financial problems can arise. This is often done even today.

One study found that women were less likely to develop excessive debt even after controlling for risk attitude, financial literacy and socio-demographic characteristics [30]. This is not what we found in the GPs’ patient population, where women were better represented than men. However, although over-indebtedness is more frequent among men, women may be more concerned about their finances. This may be related to the economic literature, which shows women to be more financially risk-averse than men [31].

There is much evidence that financial hardship and economic recessions and mediators such as unemployment, income decline and unmanageable debts are significantly associated with poor mental well-being, increased rates of common mental disorders, substance-related disorders and suicidal behaviours [32]. The question of whether health problems lead to financial problems is more difficult to answer, and the relationship will depend on the safety nets developed in various countries [2]. Norway is a welfare state and should have a strong safety net. However, this experiment and the workshop with the GPs suggested that it may be easier to control private finances if there is a specific somatic health problem rather than depression, continuous unmanaged chaotic thought patterns, an unstructured life or drug problems. Thus, the GPs should probably be more aware of the financial concerns that can result from mental illness and substance use and target those patients with information about the financial counselling services in the local labour and welfare office to reduce the risk of further health problems from continuing financial concerns.

Research results are scarce on the causal relationship between financial problems and health problems. For example, we do not know the extent to which those who struggle with major payment problems consult their GPs more often than others with the same health problems. Nor do we know whether those who have many financial concerns, perhaps without particular problems (because they have behaviours that discipline their spending), more often consult their GPs. However, our hypothesis is that financial concerns can cause mental problems, which in turn can cause financial problems, creating a risk of a downward spiral.

There is little research from Norway on the relationship between health and debt or payment problems. We have access to good data from the health registers, but until recently, available financial registers only covered secured debt (mortgages on objects). A national register of credit card debt and unsecured consumer loans was established in June 2019. However, for the time being, it is not clear whether this register will be made available for research. The comment in *The Lancet* referred to at the beginning of the paper [1] included a call to action for financial technology (Fintech) providers, health-care professionals and research scientists to work together to represent better the complementary nature of health care and finance, and that linkage of health information and individuals’ financial data could contribute to more holistic financial support systems.

This study is an attempt to lay the foundation for more advanced analyses of the relationship between health and financial problems to develop strategies to prevent people from ending up in such situations. Future research should explore the concepts of ‘debt induced illness’ and ‘illness induced debt’.

### Strengths and weaknesses

It is important to understand which actors are relevant from a prevention perspective, and this study is the first to look closely at the GP as a potential early discoverer of severe financial problems. It is a qualitative study with a small experiment with systematic mapping of the patient population in one clinic with eight GPs. We do not know whether the results would have been different if several clinics had been included or if the experiment had been conducted in another geographical area with another clinic. Furthermore, although the GPs involved in the study did not think that the reader’s letter in the local newspaper had a negative impact on the experiment, we cannot be certain that it did not.

## Conclusion

We found that 11% of patients reported financial problems that affected their health or had health problems affecting their financial situation. We suggest that GPs should ask their patients about their financial concerns when patients present with diffuse symptoms. Throughout the experiment, the GPs experienced greater awareness of financial concerns, and they found that by being more aware of this problem, they expanded and improved their toolbox. Properly used, such a tool can contribute to earlier and more correct diagnoses of patients. However, one prerequisite for success is that the financial counsellors can be reached directly on telephone numbers that the GPs give their patients. The availability and waiting time for financial counselling at the labour and welfare office is a topic that needs policy attention.

## Appendix

### About the anonymous national financial counsellor phone service

The labour and welfare services have a debt counselling phone service, with opening hours of 9.00–15.00. It is important to deal with financial problems as quickly as possible. The service gives easy advice on resolving financial situations. Callers do not need to provide their names and can be completely anonymous. The service can:

- Help people to obtain an overview of their own financial situations.
- Answer specific questions and give general advice on debt problems: debt collection, forced sale of housing, payroll, legal and extrajudicial debt settlement.
- Give advice and guidance on how to prepare for a meeting with the labour and welfare office.
- Refer to the correct service if the caller needs more help.
- Send relevant information.

The service level is good with an average waiting time of two minutes in the first three months in 2018. Most cases are about the inability to pay bills, including credit card debt/debt collection cases.
